# Supplementary material for: GP64-pseudotyped lentiviral vectors target liver endothelial cells and correct hemophilia A mice
Source: EMBO Mol Med. 2024 Apr 29;16(6):11. doi: 10.1038/s44321-024-00072-8 (PMC11178766; doi:10.1038/s44321-024-00072-8)
Supplement: Supplementary file 1 — Appendix [file 44321_2024_72_MOESM1_ESM.pdf]

GP64-pseudotyped lentiviral vectors target liver endothelial cells and correct hemophilia A mice

Michela Milani[1], Cesare Canepari[1,2], Simone Assanelli[3], Simone Merlin[3], Ester Borroni[3], Francesco Starinieri[1], Mauro Biffi[1], Fabio Russo[1], Anna Fabiano[1], Desirèe Zambroni[4], Andrea Annoni[1], Luigi Naldini[1,2], Antonia Follenzi[3], Alessio Cantore[1,2]

[1]San Raffaele Telethon Institute for Gene Therapy, IRCCS San Raffaele Scientific Institute, Milan, Italy
[2]Vita-Salute San Raffaele University, Milan, Italy
[3]Department of Health Sciences, University of Piemonte Orientale, Novara, Italy.
[4]IRCCS San Raffaele Scientific Institute, Milan, Italy

Appendix

Table of content:

Appendix Figure S1 and legend……………………………………………………………..2

Appendix Figure S2 and legend……………………………………………………………..3

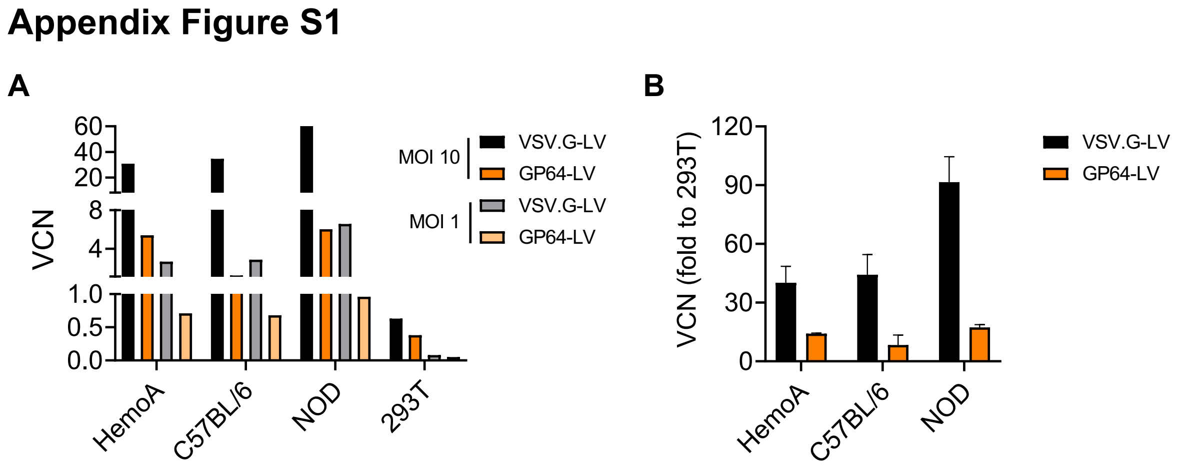

Appendix Figure S1. ***Ex vivo*** **transduction of primary murine hepatocytes**

A. VCN of primary murine hepatocytes of the indicated strains transduced with VSV.G-LV or GP64-LV at the indicated MOI. Doses have been matched based on LV titers measured on 293T cells.

B. Mean and SEM of normalized VCN values over 293T of VCN shown in A (n=2).

Appendix Figure S2

A

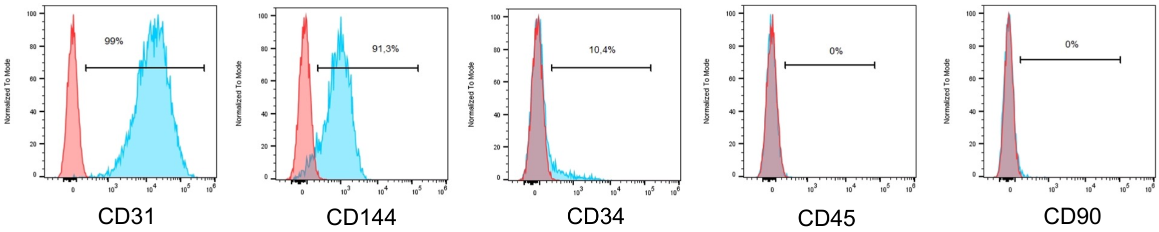

B

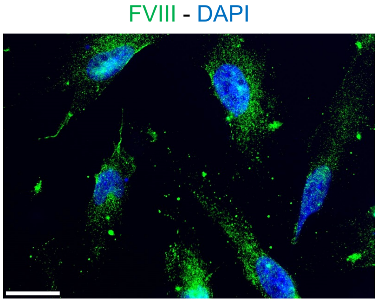

Appendix Figure S2. Characterization of immortalized LSEC

A. Flow cytometry histograms showing the expression of CD31, CD144, CD34, CD45, and CD90 in primary human immortalized LSEC.

B. Anti-human FVIII immunofluorescence image of primary human immortalized LSEC. Green: FVIII. Blue: DAPI (nuclei). Scale bar: 25µm.
