# Supplementary material for: GP64-pseudotyped lentiviral vectors target liver endothelial cells and correct hemophilia A mice
Source: EMBO Mol Med. 2024 Apr 29;16(6):11. doi: 10.1038/s44321-024-00072-8 (PMC11178766; doi:10.1038/s44321-024-00072-8)
Supplement: Supplementary file 9 — Expanded View Figures [file 44321_2024_72_MOESM9_ESM.pdf]

Expanded View Figures

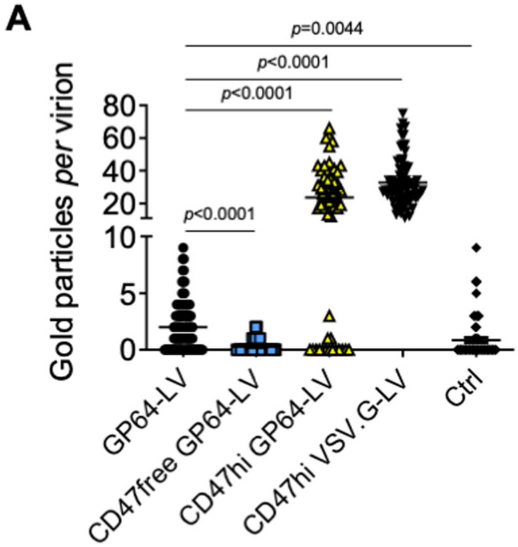

Figure EV1. Electron microscopy analysis of CD47 content on LV particles.

(**A**) Single values, mean and SEM of gold particles per virion of LV batches produced by control (GP64-LV), CD47-overexpressing (CD47hi GP64-LV or VSV.G-LV), or CD47-negative 293T cells (CD47free GP64-LV), immunostained with anti-CD47 antibody, or as staining control without the primary antibody (Ctrl) and analyzed by electron microscopy ($n = 57$–114 virions per sample). Kruskal–Wallis test with Dunn's multiple comparison test (compared to GP64-LV).

 © The Author(s)

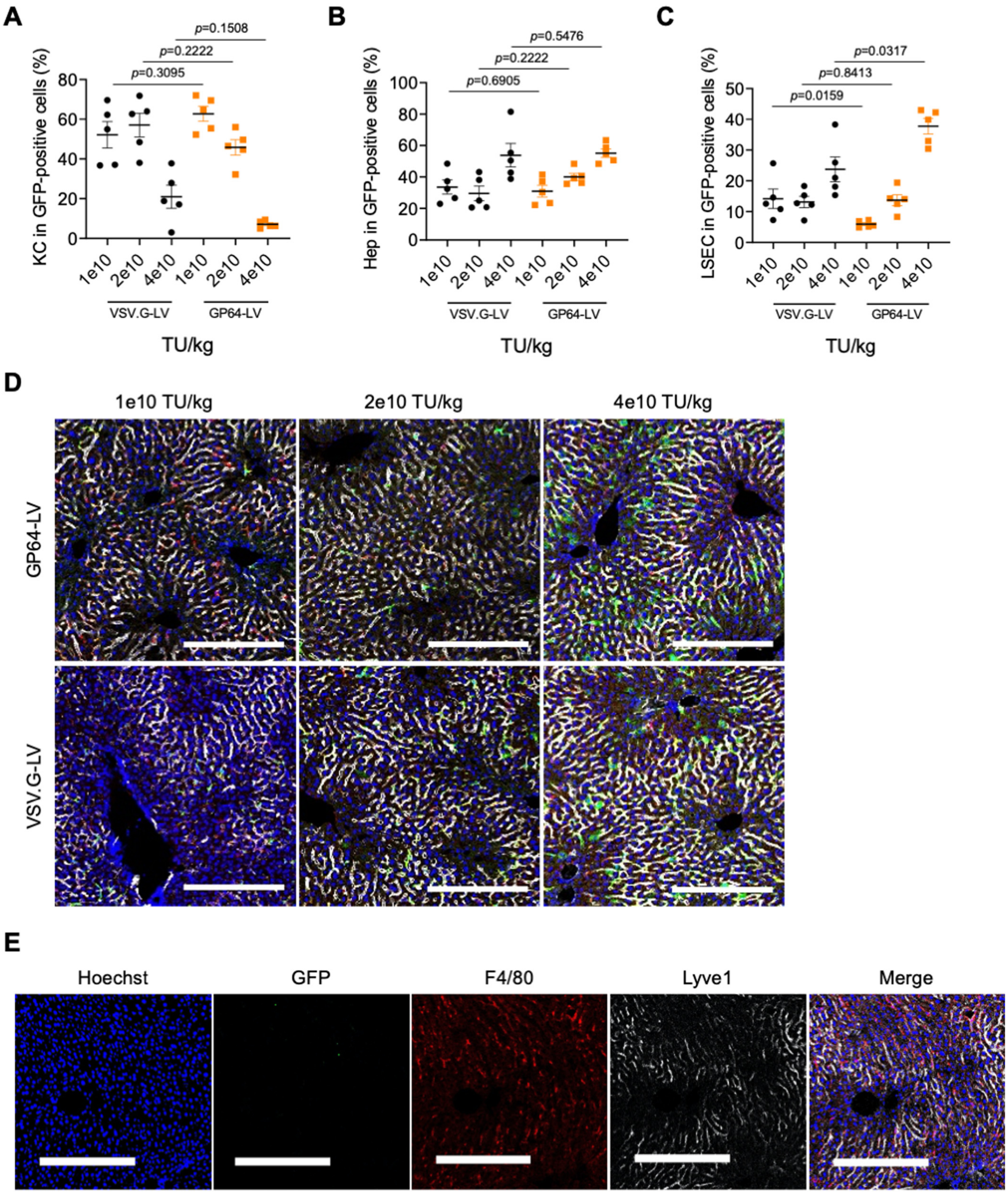
A
p=0.1508
p=0.2222
p=0.3095
KC in GFP-positive cells (%)
80
60
40
20
0
1e10
2e10
4e10
1e10
2e10
4e10
VSV.G-LV
GP64-LV
TU/kg
B
p=0.5476
p=0.2222
p=0.6905
Hep in GFP-positive cells (%)
100
80
60
40
20
0
TU/kg
C
p=0.0317
p=0.8413
p=0.0159
LSEC in GFP-positive cells (%)
50
40
30
20
10
0
TU/kg
D
1e10 TU/kg
2e10 TU/kg
4e10 TU/kg
GP64-LV
VSV.G-LV
E
Hoechst
GFP
F4/80
Lyve1
Merge

Figure EV2. Evaluation of GP64-LV liver transduction in vivo in mice.

(**A–C**) Single values, mean and SEM of the % of KC (**A**), hepatocytes (**B**) or LSEC (**C**) in GFP-positive cells shown in Fig. 3C, reported here separately for statistical analysis. $n = 5$ per group. Mann–Whitney test. (**D**). Representative merge images of immunofluorescent staining of liver sections analyzed in Fig. 3B, C at the indicated doses. Red: F4/80-positive cells (KC); White: Lyve1-positive cells (LSEC); Green: GFP (transduced cells); Blue: Hoechst (nuclei). Scale bar: 250 μm. Images of the 4e10 TU/kg LV dose are also presented in Fig. 3D. (**E**) Representative images of immunofluorescent staining of a liver section from an untreated control mouse. Red: F4/80-positive cells (KC); White: Lyve1-positive cells (LSEC); Green: GFP (transduced cells); Blue: Hoechst (nuclei). Scale bar: 250 μm.

© The Author(s)

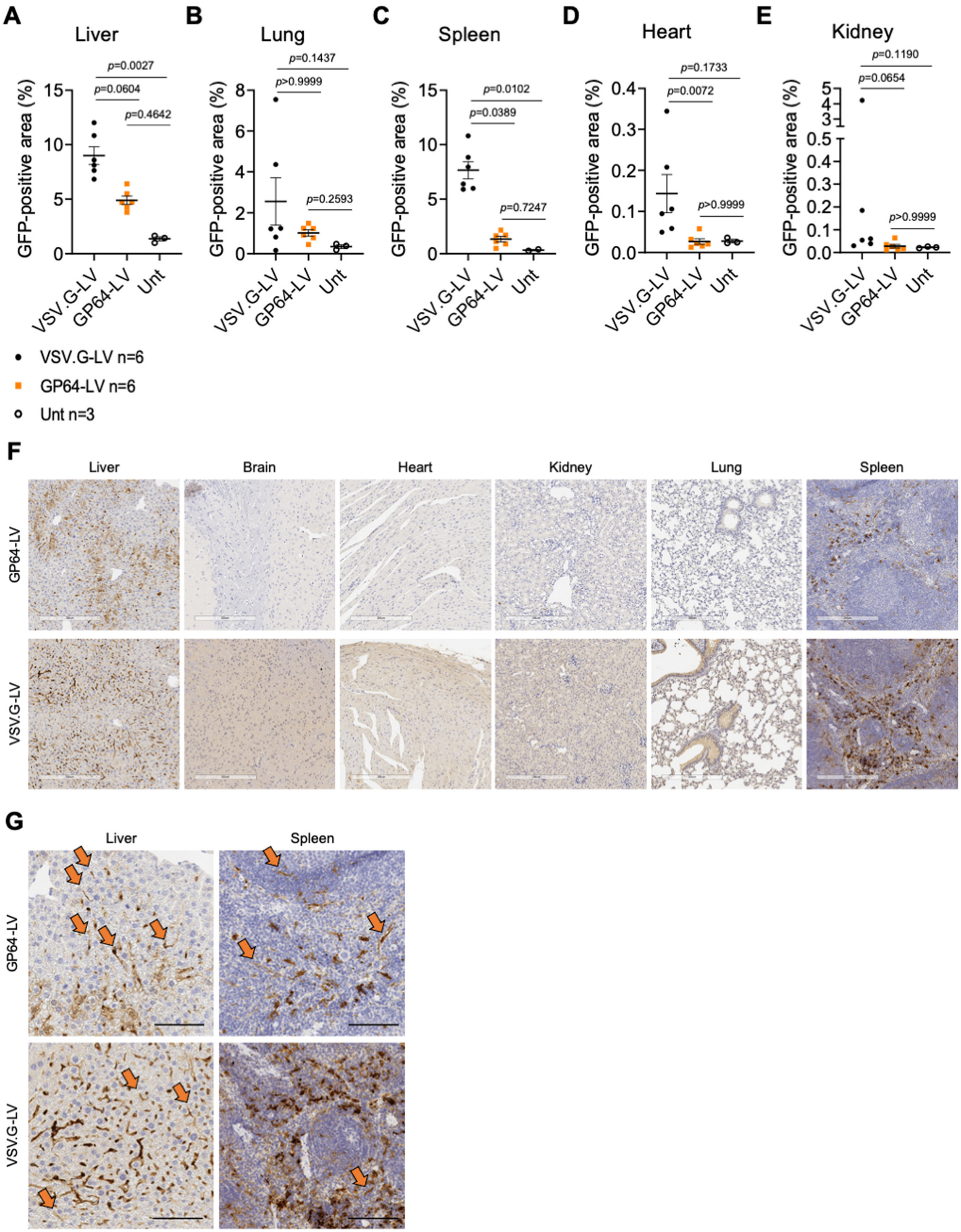
A
Liver
B
Lung
C
Spleen
D
Heart
E
Kidney
GFP-positive area (%)
p=0.0027
p=0.0604
p=0.4642
p=0.1437
p>0.9999
p=0.2593
p=0.0102
p=0.0389
p=0.7247
p=0.1733
p=0.0072
p>0.9999
p=0.1190
p=0.0654
p>0.9999
VSV.G-LV
GP64-LV
Unt
VSV.G-LV n=6
GP64-LV n=6
Unt n=3
F
Liver
Brain
Heart
Kidney
Lung
Spleen
GP64-LV
VSV.G-LV
G
Liver
Spleen
GP64-LV
VSV.G-LV

Figure EV3. Biodistribution analysis of GP64-LV after systemic administration in mice.

(**A–E**) Single values, mean and SEM of GFP-positive tissue measured by immunohistochemistry in the liver (**A**), lung (**B**), spleen (**C**), heart (**D**) or kidney (**E**) of 8-week-old mice analyzed 10 days after i.v. administration of VSV.G-LV or GP64-LV at 4e10 TU/kg ($n = 6$ mice per group), or left untreated for background signal evaluation ($n = 3$). Kruskal–Wallis test with Dunn's multiple comparison test. (**F**) Representative anti-GFP immunohistochemistry images of liver, brain, heart, kidney, lungs, spleen of mice treated with GP64-LV or VSV.G-LV, as indicated, analyzed in (**A–E**). Scale bar: 300 μm. (**G**) Higher magnification of liver and spleen sections shown in (**F**). Orange arrows indicate GFP-positive endothelial cells, identified by morphology. Scale bar: 100 μm.

 © The Author(s)

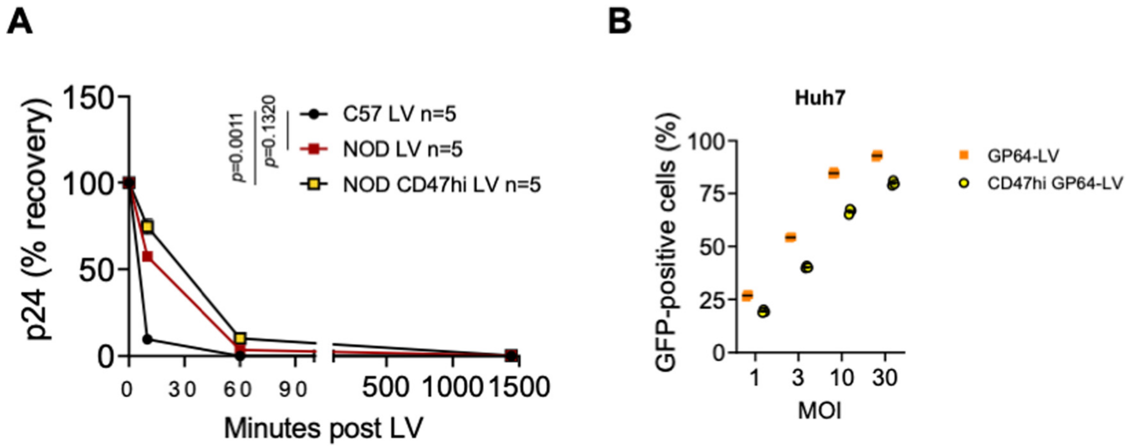

Figure EV4. GP64-LV half-life after systemic administration in mice.

(**A**) Percentage of the serum concentrations of LV particles (measured as HIV Gag p24) recovered at the indicated time (minutes) after administration of GP64-LV or CD47hi GP64-LV relative to the total amount of administered LV particles to C57BL/6 or NOD mice, as indicated (8e10 TU/kg). Kruskal–Wallis test with Dunn's multiple comparison test (compared to C57 LV). (**B**) Single values, mean, and SEM of the percentage of GFP-positive Huh7 cells, transduced with GP64-LV or CD47hi GP64-LV, at the indicated MOI ($n = 3$).

© The Author(s)

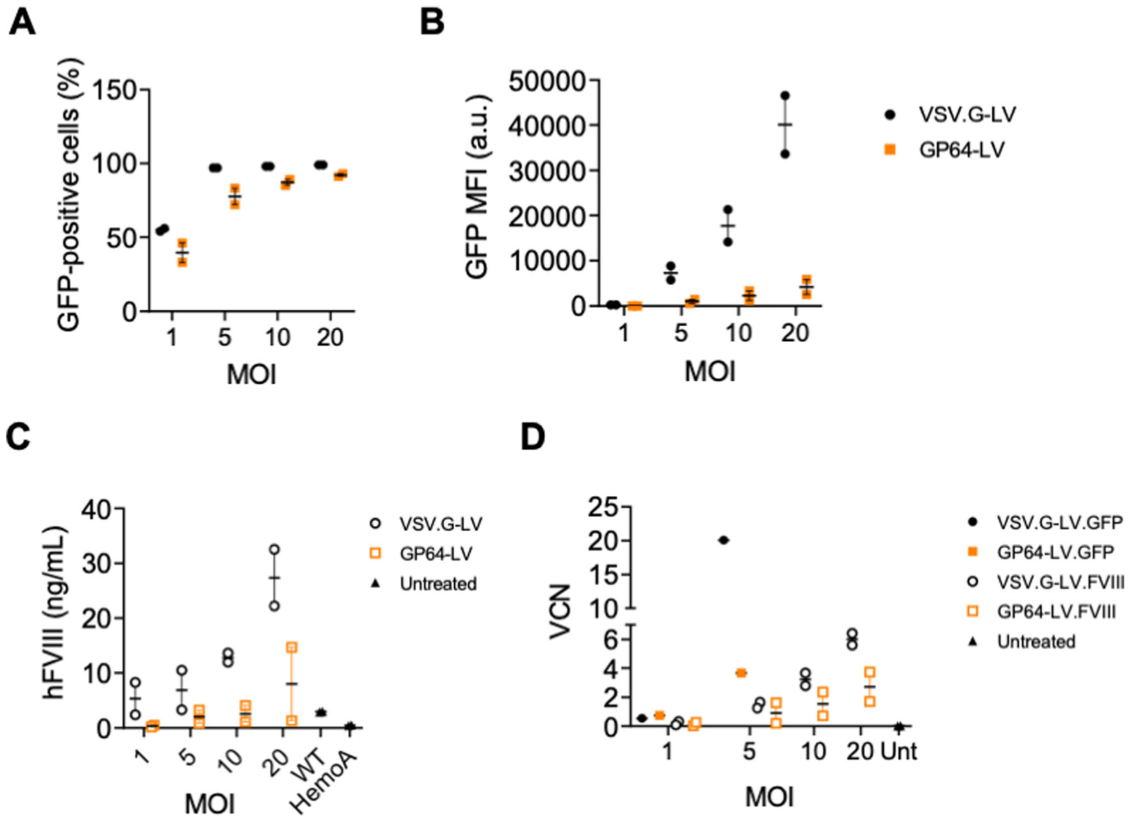

Figure EV5. Ex vivo transduction of primary human blood outgrown endothelial cells.

(**A**, **B**) Mean and SEM with single values of the % (**C**) or MFI (**D**) of GFP-positive primary blood outgrowth endothelial cells (BOEC) derived from a hemophilia A donor ($n = 2$ independent experiments) transduced with VSV.G-LV or GP64-LV at the indicated MOI. (**C**) Mean and SEM with single values of the amount of hFVIII measured in the culture supernatant of primary BOEC derived from a hemophilia A donor ($n = 2$ independent experiments) transduced with VSV.G-LV or GP64-LV at the indicated MOI, or left untreated. hFVIII concentration reported is obtained from two supernatant collection. (**D**) Mean and SEM with single values of the VCN of reversed transcribed LV genome of the primary BOEC (shown in (**A**–**C**)) transduced with VSV.G-LV or GP64-LV encoding for GFP or hFVIII at the indicated MOI, or left untreated.

 © The Author(s)
